# Morphometric and signal intensity benchmarks of 3D CRANI MR neurography sequence for extraforaminal cranial and occipital nerves visualization: a pilot study

**DOI:** 10.1007/s00276-025-03726-5

**Published:** 2025-09-29

**Authors:** Iraj Ahmadzai, Frederic Van der Cruyssen, Sohaib Shujaat, Jan W. Casselman, Constantinus Politis, Reinhilde Jacobs

**Affiliations:** 1 Department of Oral and Maxillofacial Surgery, University Hospitals Leuven, Leuven, Belgium; 2 OMFS-IMPATH Research Group, Department of Imaging and Pathology, Faculty of Medicine, University Leuven, Leuven, Belgium; 3 Department of Maxillofacial Surgery and Diagnostic Sciences, King Abdullah International Medical Research Center, College of Dentistry, King Saud Bin Abdulaziz University for Health Sciences, Ministry of National Guard Health Affairs, Riyadh, Kingdom of Saudi Arabia; 4 Dalhousie University, Halifax, NS Canada; 5 Department of Oral Health Sciences, KU Leuven and Department of Dentistry, University Hospitals Leuven, Leuven, Belgium; 6 Department of Dental Medicine, Karolinska Institutet, Nobels Väg 6, 171 77 Stockholm, Sweden

**Keywords:** MRI, Magnetic resonance neurography, Head and neck imaging, Cranial nerves, Occipital nerve

## Abstract

**Purpose:**

Lack of evidence exists related to the reporting of benchmark values of MR neurography sequences required for extraforaminal cranial and occipital nerve visualization. This study aimed to establish benchmarks of morphometric and signal intensity values of 3D CRANI MR neurography sequence in healthy subjects.

**Methods:**

A total of 10 healthy participants (5 males, 5 females; age range:14–83 years) were recruited. Imaging was conducted using a 3.0 Tesla MRI system fitted with a 32-channel head coil. The assessed extraforaminal cranial and occipital nerves included auriculotemporal, buccal, facial, greater occipital, hypoglossal, inferior alveolar, lingual, mandibular, masseteric, and maxillary. These nerves were semi-automatically segmented and divided into five segments: proximal, mid-proximal, middle, mid-distal, and distal. Measurements were performed for per nerve and segment diameter, signal intensity, apparent signal-to-noise (aSNR) and apparent nerve-muscle contrast-to-noise ratios (aNMCNR).

**Results:**

All nerves exhibited a decreasing trend in diameter and signal intensity from the proximal to the distal end, except for the facial, maxillary, and auriculotemporal nerves. The mid-proximal section of the nerves under examination showed notably higher values for diameter (*p* < 0.01), signal intensity (*p* < 0.0001), and aNMCNR (*p* < 0.05). On the other hand, the distal segment recorded the lowest values across all parameters. The aSNR and aNMCNR values confirmed good discrimination of each observed nerve.

**Conclusions:**

The proposed benchmark for 3D CRANI MR neurography enhances the neuroradiological understanding of cranial and occipital nerves. It could act as a reference guide in various head and neck scenarios, particularly when distinguishing between healthy and pathological conditions.

**Supplementary Information:**

The online version contains supplementary material available at 10.1007/s00276-025-03726-5.

## Introduction

The cranial and occipital nerves, crucial for the human body’s functionality, cater to the intricate anatomical region of the head and neck. These nerves, with their complex network of fibers, enable signal transmission between the brain and various parts of the head and neck. They regulate a wide array of functions, from sensory perception in the face to motor control in the neck muscles. Peripheral neuropathy can occur when these nerves are damaged during various surgical procedures, including mandibular wisdom tooth extraction, dental implant placement, orthognathic surgery, and neurosurgery [7]. Additional factors contributing to neuropathy encompass chemotherapy and diabetes [1, 10].

Currently, the gold standard for diagnosing peripheral neuropathies involves clinical neurosensory testing (NST). However, due to ongoing postoperative changes and methodological challenges, NST data may be unreliable. For instance, in the months immediately following nerve damage, the nerves undergo a recovery process. This phase is characterized by significant fluctuations in sensory perception, which can render the outcomes of NST unreliable [5]. Symptoms such as brush stroke allodynia, thermal and/or mechanical hyperesthesia, along with the extent of the neuropathic area, can have a profound impact on an individual’s subjective well-being. It is also important to highlight that the median time span between nerve injury and initial examination in certain studies is approximately 1.6 months [5]. These elements can add complexity and inconsistency to the interpretation of testing. Moreover, NST is unable to accurately identify the precise location of injury or outline the anatomy for presurgical planning if microsurgical repair is contemplated [5]. Although ultrasound has been proposed as a promising technique for diagnosing peripheral nerve disorders, it is also prone to certain limitations. These include low resolution, technical challenges in examining long limb nerves running in various angles and directions, lack of sufficient anatomic detail, and potential discomfort to the patient during extensive examinations. Similarly, routine MR imaging sequences fail to provide a reliable visualization of peripheral nerves [8].

Until recently, the advancements in MR imaging techniques, such as MR neurography with dedicated sequences, have now permitted enhanced visualization of peripheral nerves. Such sequences could potentially serve as a valuable diagnostic tool in patients with peripheral neuropathy. Previous studies have demonstrated that through the application of 3D CRANI MR neurography sequence, extraforaminal cranial and occipital nerves can be reliably visualized [3, 14]. However, a deeper understanding of the radioanatomy of nerves in both physiological and pathological states is essential for its clinical implementation. Currently, there is a lack of evidence pertaining to the documentation of reference morphometric and signal intensity values for 3D CRANI MR neurography of peripheral cranial and occipital nerves.

Therefore, the current study aimed to quantitatively benchmark the morphometric and signal intensity values of selected extraforaminal cranial and occipital nerves using the 3D CRANI MR neurography sequence in healthy subjects. The selection of nerves was guided by their consistent visualization using the 3D CRANI sequence and their clinical importance.

## Materials and methods

This observational study was conducted in adherence to the STROBE guidelines and Declaration of Helsinki. Ethical approval (reference number: S61077) was acquired from the Ethics Committee of University Hospitals, KU Leuven, Leven, Belgium. In addition, informed consent was obtained from all participants. A total of ten consecutive healthy participants (5 males, 5 females; average age: 47 years; age range: 14–83 years) who had undergone 3D CRANI MR neurography during the study period were included based on image quality and availability of informed consent. Inclusion criteria required healthy volunteers without any history of neuropathy, head or neck surgery, trauma, or neurological disorders. The exclusion criteria were thus volunteers with a history of neuropathy, head or neck surgery, trauma, or neurological disorders. The selection of nerves was based on their expected visibility using the CRANI sequence, their frequent involvement in clinical pathologies, and their inclusion in earlier feasibility studies [6, 7].

### Image acquisition

The imaging was conducted using a 3.0 Tesla MRI system (Ingenia; Philips, Best, Netherlands), equipped with a 32-channel head coil (INVIVO, Gainesville, USA). A previously established MR neurography sequence, known as 3D CRANI, was utilized [14]. The 3D CRANI is a 3D short inversion time inversion-recovery (STIR) turbo spin–echo (TSE) sequence that employs a pseudo-steady state (PSS) sweep in conjunction with Motion Sensitized Driven Equilibrium (MSDE) pulse. To ensure uniform signal suppression from muscle, fat, and blood across the field of view, STIR was used in conjunction with MSDE. The 3D CRANI sequence was initiated immediately following the bolus injection of gadolinium, which was administered at a rate of 3 cc per second.

The parameters applied for imaging were as follows: repetition time (TR) = 2300, echo time = 188, field of view = 200 × 200 × 90 mm, slice thickness = 0.9 mm, actual slice gap = − 0.45, matrix = 224 × 222, acquired voxel size = 0.9 × 0.9 × 0.9 mm, reconstructed voxel size = 0.6 × 0.6 × 0.45 mm, slice oversampling = 1.5, compressed sense-reduction = 2, number of slices = 200,TSE factor = 43 (startup echoes 2), number of acquisitions = 1, scanning time = 8:08 min, black blood pulse = MSDE [flow ghost suppression].

### Imaging analysis, data collection and measurements

**Fig. 1:**
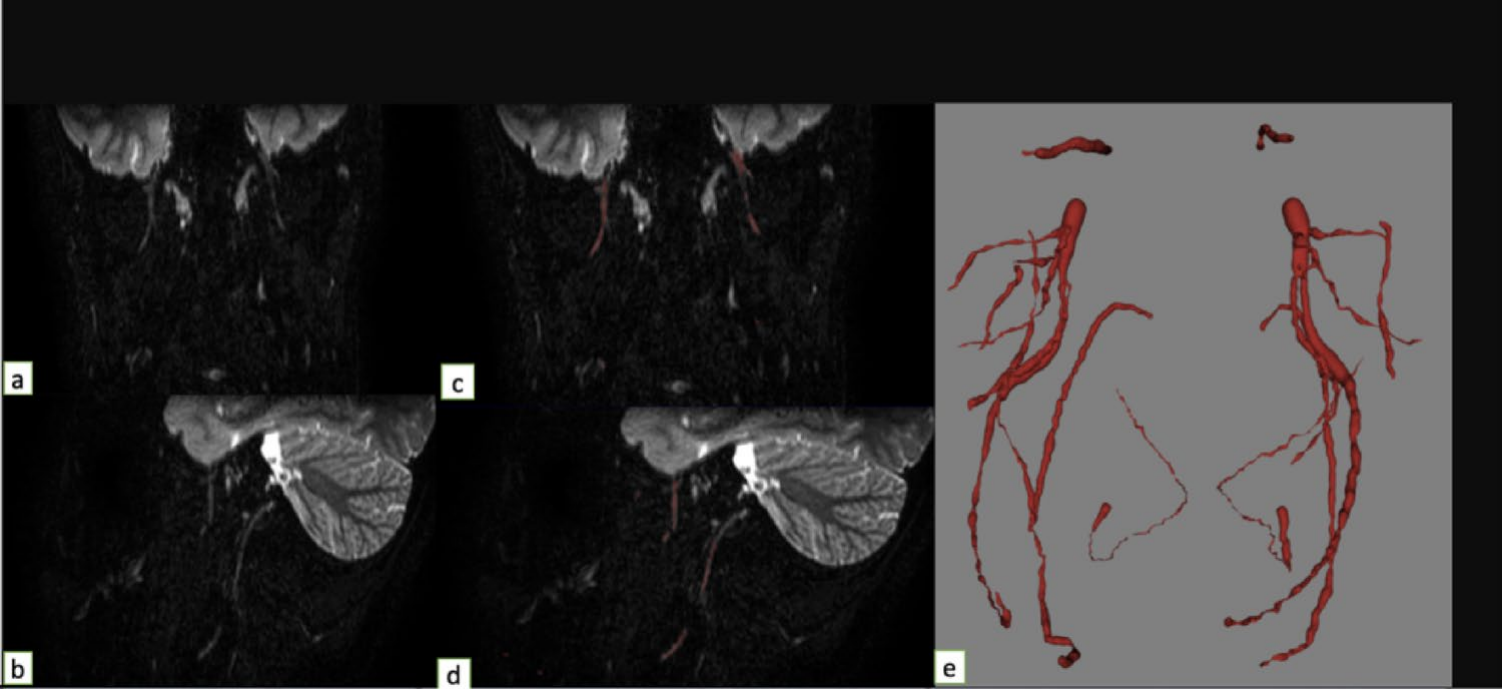
**a** Unsegmented coronal MRN slice. **b** Unsegmented sagittal MRN slice. **c** Segmented coronal MRN slice. **d** Segmented sagittal MRN slice. **e** Overview of segmented nerves as shown in segmentation software (Relu)

**Fig. 2:**
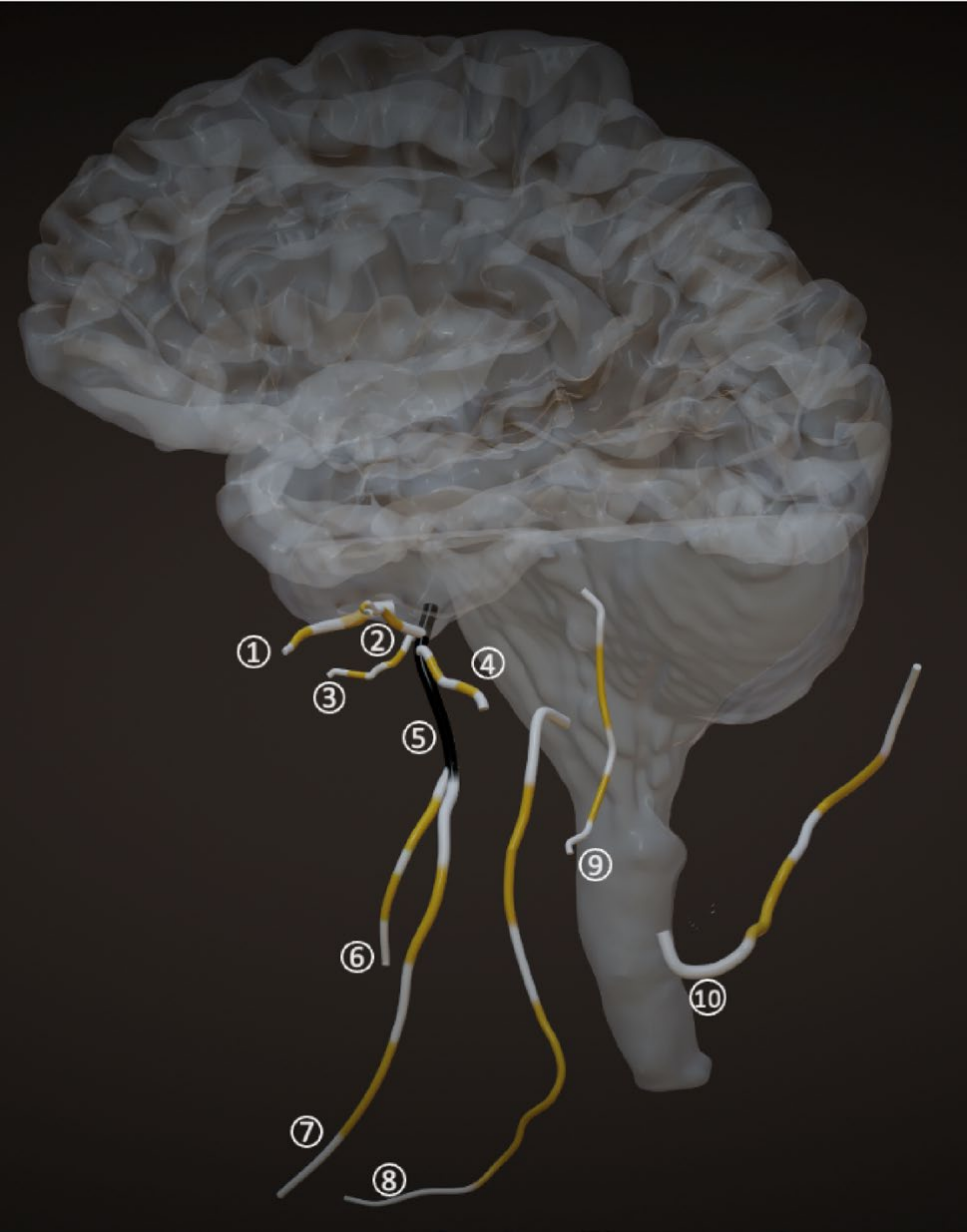
Sagittal plane of 3D map drawn based on semi-automated segmentations of following nerves (1: Maxillary, 2: Masseteric, 3: Buccal, 4: Auriculotemporal, 5: Mandibular, 6: Lingual, 7: Inferior alveolar, 8: Hypoglossal, 9: Facial, 10: Greater occipital)

**Fig. 3:**
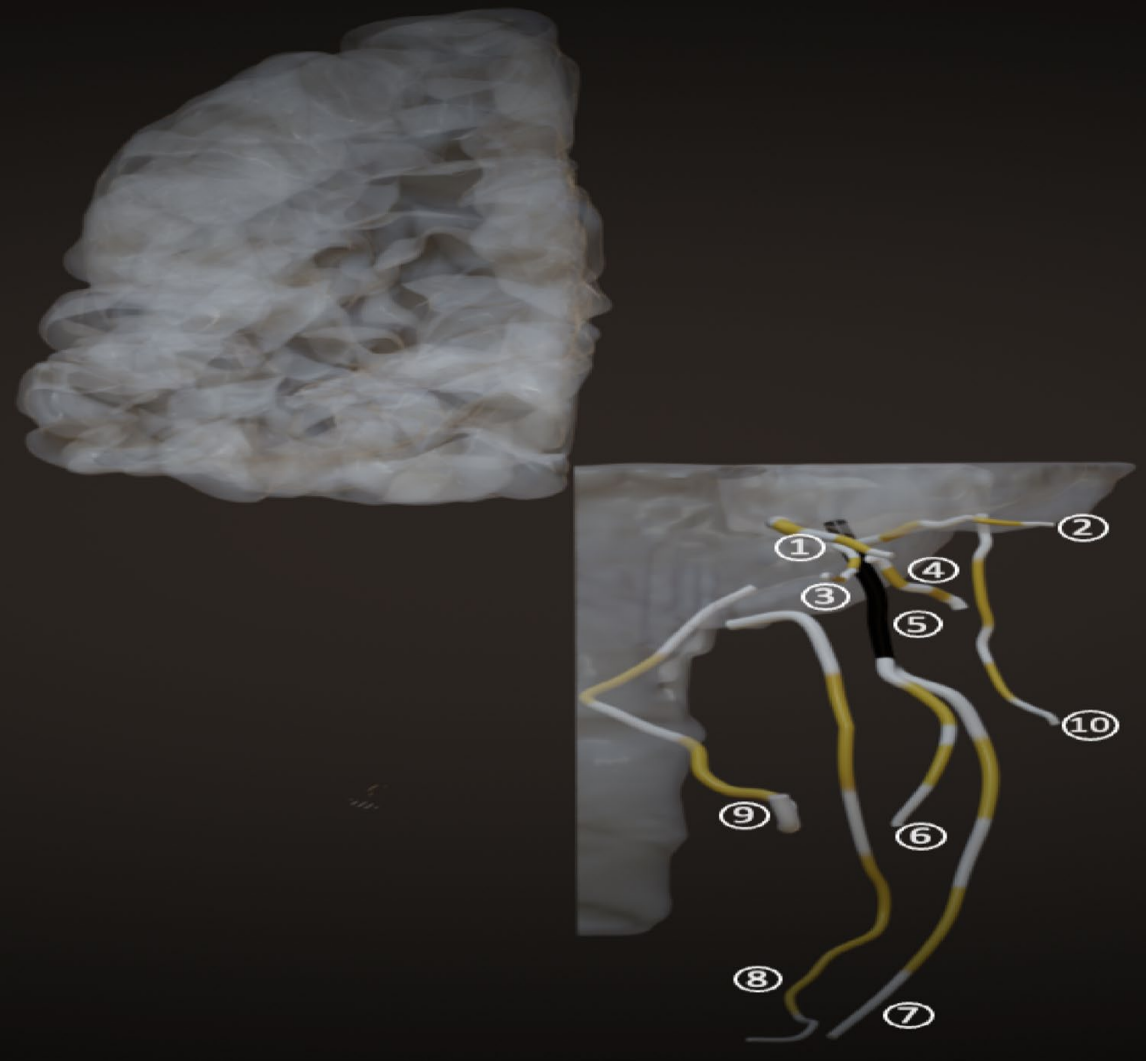
Frontal plane of 3D map drawn based on semi-automated segmentations of following nerves: (1: Maxillary, 2: Masseteric, 3: Buccal, 4: Auriculotemporal, 5: Mandibular, 6: Lingual, 7: Inferior alveolar, 8: Hypoglossal, 9: Facial, 10: Greater occipital)

Prior to the commencement of data collection, all neurography images were anonymized. The following nerves were analyzed: auriculotemporal, buccal, facial, greater occipital, hypoglossal, inferior alveolar, lingual, mandibular, masseteric, and maxillary. Two independent observers identified the nerves on the images. The segmentation of cranial and occipital nerves was performed using a semi-automated approach within the Relu software environment (www.relu.eu, Leuven, Belgium), a dedicated 3D medical imaging platform. Nerves were segmented along their full visible course using a thresholding-based algorithm integrated in the Relu software. This method allowed for consistent delineation of nerve boundaries across cases while minimizing variability. Segmented nerves were then reconstructed into three-dimensional virtual models (Fig. 1). Four anatomical reference points were placed per nerve to divide each into five segments—proximal, mid-proximal, middle, mid-distal, and distal—enabling systematic analysis (Figs. 2 and 3). Following segmentation, the Relu software automatically extracted quantitative values for nerve diameter, signal intensity, apparent signal-to-noise ratio (aSNR), and apparent nerve-muscle contrast-to-noise ratio (aNMCNR) based on grey-scale maps of the segmented structures. This automated extraction process ensured standardized and reproducible measurement of morphometric and signal-related parameters across all subjects.

### Statistical analysis

Data were analyzed using RStudio version 1.2.5001 (RStudio Team [2020]. RStudio: Integrated Development for R. RStudio, PBC, Boston, MA. http://www.rstudio.com/). Following the pooling of data from the left and right sides, descriptive statistics were performed. The continuous measurements taken along each nerve trajectory were divided into five segments: proximal, mid-proximal, middle, mid-distal, and distal. Subsequently, nerve diameter and signal intensity boxplots were plotted and stratified per nerve and segment. Both non-normalized and normalized values were computed to facilitate future comparisons, as per earlier described method [3]. To compare continuous measurements across groups, ANOVA tests were employed. A *p* value of < 0.05 was considered as statistically significant.

## Results

To support the interpretation of the results and anatomical understanding, Table 1 provides a schematic summary of the anatomical course of the selected cranial and. Occipital nerves as assessed in this study [11, 12].

**Table 1:** Anatomical course of selected extraforaminal cranial and occipital nerves

	Origin	Foramen	Extracranial course	Function
Auriculotemporal nerve	Posterior division of V3	N/A	Encircles middle meningeal artery; ascends anterior to ear	Sensory to temple, auricle, and TMJ
Buccal nerve	Anterior division of V3	N/A	Passes between lateral pterygoid and buccinator muscles	Sensory to cheek mucosa and skin
Facial nerve	Pontomedullary junction	Stylomastoid foramen	Traverses parotid gland and splits into five main branches (temporal, zygomatic, buccal, mandibular, cervical)	Motor innervation of facial muscles; critical in parotid and facial surgery
Greater occipital nerve	Dorsal ramus of C2	Between C1 and C2 vertebrae	Pierces semispinalis capitis and trapezius to reach posterior scalp	Sensory to posterior scalp; common in occipital neuralgia
Hypoglossal nerve	Medulla oblongata	Hypoglossal canal	Courses beneath digastric, crosses internal carotid artery and vagus nerve to reach tongue	Motor to all intrinsic and extrinsic tongue muscles
Inferior alveolar nerve	Posterior division of V3	Mandibular foramen	Travels within mandibular canal; exits as mental nerve	Sensory to lower teeth and chin
Lingual nerve	Posterior division of V3	N/A	Courses medial to mandibular ramus towards tongue	Sensory innervation to anterior two-thirds of tongue
Mandibular nerve	Trigeminal ganglion (CN V)	Foramen ovale	Branches into buccal, lingual, auriculotemporal, masseteric, and inferior alveolar nerves	Mixed motor-sensory; relevant in jaw surgery and nerve injuries
Masseteric nerve	Anterior division of V3	Indirect via foramen ovale	Traverses mandibular notch to reach masseter muscle	Motor to masseter muscle
Maxillary nerve	Trigeminal ganglion (CN V)	Foramen rotundum	Passes through pterygopalatine fossa and infraorbital canal; exits via infraorbital foramen	Sensory innervation of midface

N/A, not applicable

**Fig. 4:**
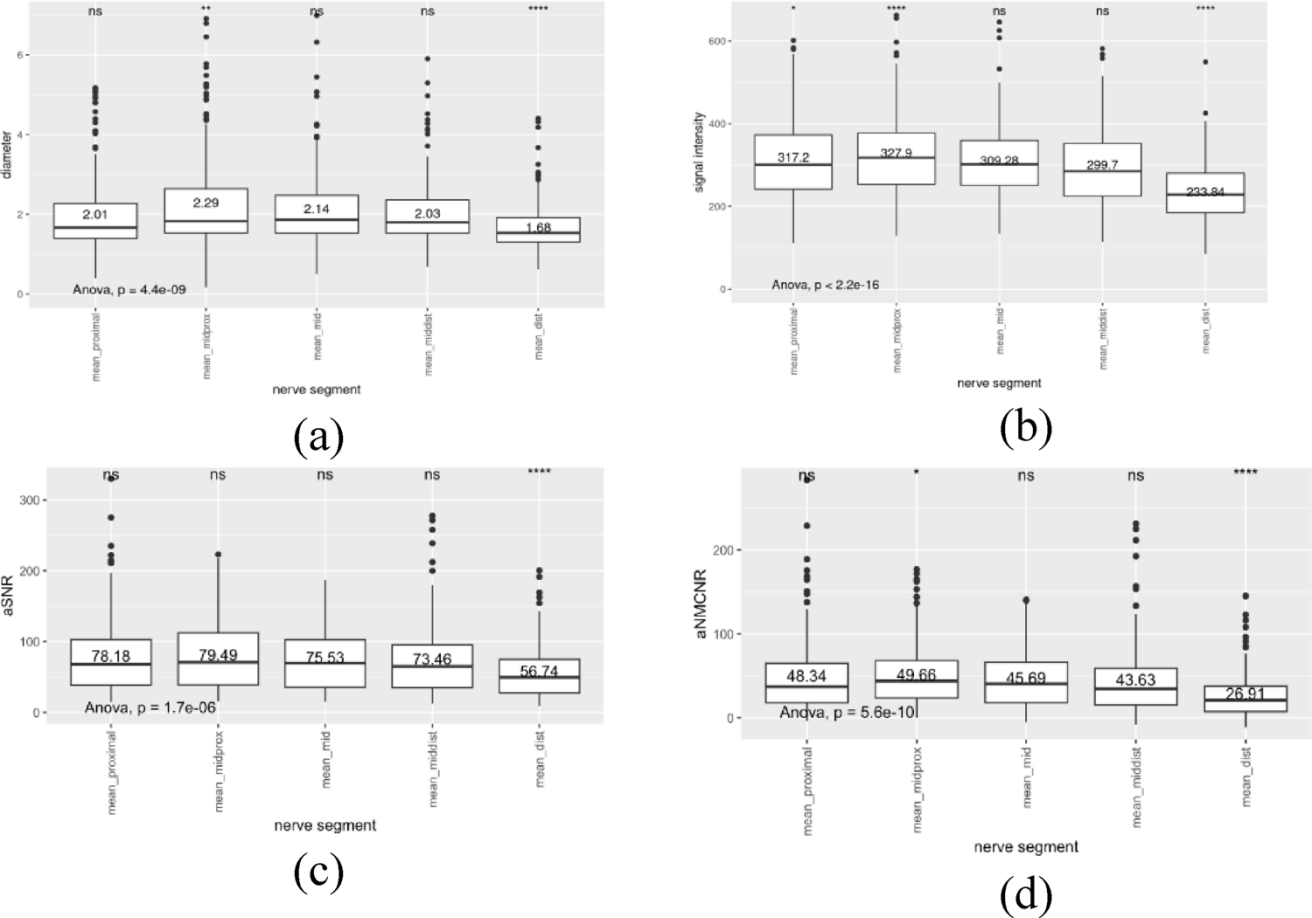
Boxplots of mean diameter, mean signal intensity, mean aSNR and mean ANMCNR for all nerves stratified per segment. (ns: not significant; *p < 0.05; **p < 0.01; ***p < 0.001;****p < 0.0001). **a** Boxplots of mean diameters for all nerves stratified per segment. **b** Boxplots of mean signal intensity for all nerves stratified per segment. **c** Boxplots of aSNR for all nerves stratified per segment. 4d Boxplots of aNMCNR for all nerves stratified per segment

The boxplots of mean diameter, signal intensity, aSNR, and aNMCNR (Fig. 4) revealed that the mid-proximal segment generally exhibited the highest values (diameter: 2.3 mm, intensity: 327.9, aSNR: 79.5, aNMCNR: 49.7). These values significantly differed in terms of mean diameter (*p* < 0.01), mean signal intensity (*p* < 0.0001), and aNMCNR (*p* < 0.05). In contrast, the distal segment displayed the lowest values (diameter: 1.7 mm, intensity: 233.8, aSNR: 56.7, aNMCNR: 26.9), with significant differences observed for all measured parameters (*p* < 0.0001). The proximal segment only exhibited a significant difference in mean signal intensity (*p* < 0.05).

A consistent reduction in diameter was noted for all nerves, excluding the facial, maxillary, and auriculotemporal nerves. This gradual decrease in nerve diameter ranged from 1.86 mm to 0.1 mm. The mandibular and greater occipital nerves demonstrated the most and least notable reduction, respectively. The facial, auriculotemporal, and maxillary nerves exhibited an increase in diameter of 0.26 mm, 0.14 mm, and 0.10 mm, respectively (Table 2). Nerve diameters as small as 0.17 mm were observable.

**Table 2:** Mean diameter (mm) stratified per segment and per nerve

	Proximal	Midproximal	Middle	Middistal	Distal
Auriculotemporal nerve	1.23	1.50	1.66	1.64	1.37
Buccal nerve	1.44	1.62	1.66	1.70	1.40
Facial nerve	1.74	1.92	1.90	2.03	2.00
Greater occipital nerve	1.56	1.52	1.44	1.53	1.46
Hypoglossal nerve	1.60	1.53	1.55	1.69	1.41
Inferior alveolar nerve	2.49	2.73	2.33	2.17	1.62
Lingual nerve	1.78	2.17	1.98	1.70	1.45
Mandibular nerve	4.42	5.05	4.08	3.38	2.56
Masseteric nerve	1.59	1.79	1.83	1.73	1.36
Maxillary nerve	1.91	2.57	2.48	2.44	2.01

**Fig. 5:**
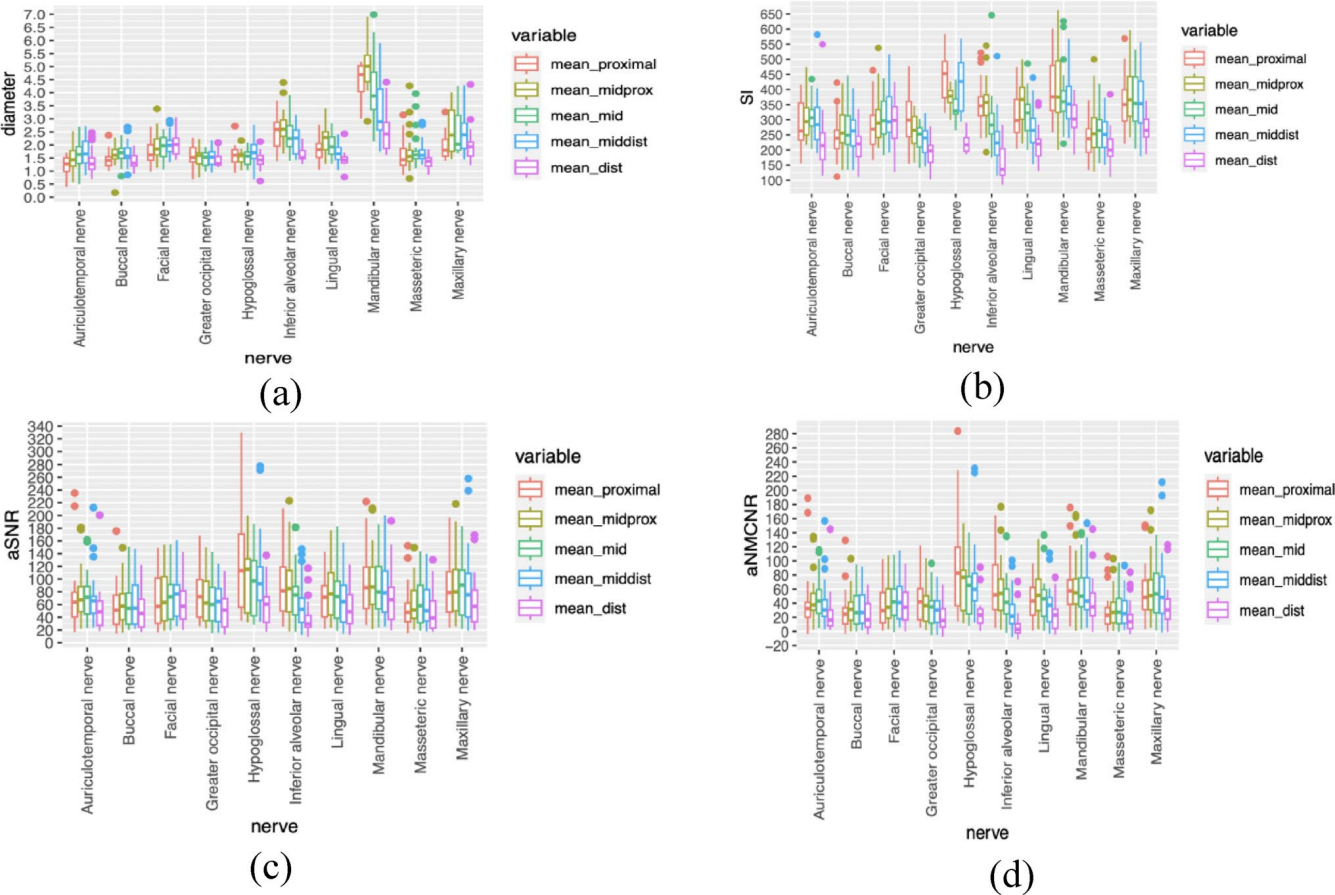
Boxplots of mean diameter, mean signal intensity, mean aSNR and mean ANMCNR stratified per nerve and per segment. **a** Boxplots of mean diameter stratified per nerve and per segment. **b** Boxplots of signal intensity stratified per nerve and per segment. **c** Boxplots of aSNR stratified per nerve and per segment. **d** Boxplots of aNMCNR stratified per nerve and per segment

A decline in signal intensity from proximal to distal segment was observed across all nerves, with the exception of facial nerve. The most substantial and the least significant signal drop occurred in the hypoglossal nerve and buccal nerve, with a decrease of 210.8 and 28.5, respectively. The facial nerve exhibited an increased signal intensity of 14.5 (Table 3). The aSNR (Table 4) and aNMCNR values (Table 5) confirmed good discrimination of each of the observed nerves. The facial nerve was the only nerve that did not show a gradual drop in aSNR and aNMCNR (Fig. 5).

**Table 3:** Mean signal intensity (non-normalized) stratified per segment and per nerve

	Proximal	Midproximal	Middle	Middistal	Distal
Auriculotemporal nerve	274.09	300.88	309.19	292.37	227.96
Buccal nerve	234.05	254.59	260.70	258.56	213.85
Facial nerve	277.04	298.29	291.76	302.18	279.69
Greater occipital nerve	298.93	265.54	239.57	232.47	194.87
Hypoglossal nerve	443.19	373.44	338.54	418.61	222.41
Inferior alveolar nerve	358.54	350.55	303.74	244.53	154.26
Lingual nerve	306.06	350.05	309.38	265.45	212.49
Mandibular nerve	384.16	386.35	364.15	345.35	293.76
Masseteric nerve	231.23	258.36	266.49	258.17	206.26
Maxillary nerve	342.98	370.92	362.95	355.02	273.90

**Table 4:** Mean aSNR stratified per segment and per nerve

	Proximal	Midproximal	Middle	Middistal	Distal
Auriculotemporal nerve	72.43	75.88	76.31	71.74	54.77
Buccal nerve	58.45	62.88	63.32	61.90	51.61
Facial nerve	66.24	71.83	71.51	72.60	66.45
Greater occipital nerve	76.19	68.62	64.75	61.98	52.70
Hypoglossal nerve	129.46	102.39	93.30	115.72	61.15
Inferior alveolar nerve	89.91	88.95	76.07	61.02	39.05
Lingual nerve	72.58	84.24	75.34	65.79	52.53
Mandibular nerve	92.75	93.72	88.30	84.95	72.75
Masseteric nerve	57.53	62.20	62.53	59.86	49.36
Maxillary nerve	84.53	89.30	86.95	90.15	66.48

**Table 5:** Mean aNMCNR stratified per segment and per nerve

	Proximal	Midproximal	Middle	Middistal	Distal
Auriculotemporal nerve	42.46	45.91	46.34	41.77	24.80
Buccal nerve	29.25	33.68	34.12	32.70	22.40
Facial nerve	37.03	42.63	42.31	43.40	37.25
Greater occipital nerve	44.68	37.11	33.24	30.47	21.20
Hypoglossal nerve	95.89	68.82	59.73	82.15	27.58
Inferior alveolar nerve	59.94	58.98	46.09	31.05	9.08
Lingual nerve	43.38	55.04	46.14	36.59	23.33
Mandibular nerve	63.54	64.52	59.09	55.75	43.55
Masseteric nerve	28.32	33.00	33.33	30.66	20.16
Maxillary nerve	55.32	60.10	57.75	60.94	37.28

## Discussion

At present, there is a noticeable gap in the scientific literature regarding the benchmark values for either physiological or pathological 3D CRANI MR neurography sequence pertaining to cranial and occipital nerves visualization. Establishing quantitative metrics of intact nerves along their proximo-distal course represents an initial step towards a dependable and efficient quantitative morphological evaluation of peripheral neuropathy. Hence, the following study established the benchmark values for diameter and signal intensity of nine different cranial nerves or branches thereof, as well as the greater occipital nerve using a previously developed 3D CRANI MR neurography sequence. Both normalized and non-normalized values were calculated and presented to allow for future comparative analysis with pathological cases.

The findings of the present study suggested a decrease in signal intensity of the majority of nerves, extending from the proximal to distal segment. As anticipated, the diameter of the nerve also diminished distally, a finding that has been corroborated by evidence related to anatomical dissection of the nerves [6, 15]. Moreover, prior studies identified similar alterations in signal patterns of healthy individuals [2–5]. In contrast, facial, maxillary, and auriculotemporal nerves showed an increase in diameter from the proximal to distal end. This observation may be attributed to measurement artifacts. Signal suppression of surrounding fat, vessels, and glands by the 3D CRANI sequence can blur anatomical boundaries, leading to partial volume effects and overestimation during segmentation. In line with this, Salame et al. reported the diameter of the facial nerve trunk at the stylomastoid foramen to be 2.66 ± 0.55 mm, which aligns with the upper range of our facial nerve values [13]. Regarding aSNR values, the generally high ratios across most nerves reflect the sequence’s capacity to isolate neural signal from surrounding tissues. In a clinical context, high aSNR is essential for visualizing small-caliber nerves and distinguishing them from vascular or muscular structures. A drop in aSNR in distal segments may suggest signal attenuation due to decreased nerve caliber or partial volume effects. These findings are vital for enabling further interpretation of nerve trajectories and for identifying pathological abnormalities in cranial and occipital neuropathy. Hence, further lending credence to the practicality of employing MR neurography sequences specifically tailored for nerves. Such a 3D sequence augments the existing body of knowledge in the field of neuroradiology, particularly for visualizing and investigating peripheral nerves.

The main strength of this study lies in its first-time reporting of benchmark values for morphometry and signal intensities of both the entire and segmented sections of extraforaminal cranial and occipital nerves, using a dedicated MR sequence. Furthermore, the research included a broad age range and an equal gender distribution, which could facilitate a more comprehensive understanding of normal variations. Simultaneously, the research was subject to certain limitations. The sample size was relatively small, and there was a limited number of observers, which could have potentially resulted in measurement bias. Consequently, one should exercise prudence while interpreting the results.

It is recommended to conduct future studies with a greater sample size to externally validate the 3D CRANI sequence in both healthy and pathological instances. Studies with a greater sample size may also explore potential anatomical or signal-related differences between male and female subjects. Furthermore, additional parameters such as the length of the segmented nerves could be included in future studies to enhance the morphometric characterization. A comparison of its diagnostic yield with other MR neurography techniques could provide valuable insights for clinical and scientific purposes. An intriguing area of exploration would be the impact of fat suppression and T2 signal intensity when applying the 3D CRANI sequence. Given that this sequence is heavily T2-weighted and fat-suppressed, the lipid-rich myelin sheaths that encase most cranial nerves are expected to be significantly suppressed [9]. Conversely, a high signal intensity is anticipated from axonal fluid. It would be worthwhile to examine how these two factors interact by studying the effect of myelinated versus unmyelinated nerves on signal intensity in a future study. Furthermore, it would be interesting to investigate how this might influence patients with demyelinating disease states and how it correlates with electrophysiological examinations.

## Conclusion

The presented benchmark morphometric and signal intensity values of extraforaminal cranial and occipital nerves using 3D CRANI MR sequence could serve as a reference guide for diagnosis and treatment planning in neuropathic conditions. It is imperative for radiologists and clinicians to familiarize themselves with such MRN sequences to facilitate the exploration of potential clinical applications.

## Supplementary Information

Below is the link to the electronic supplementary material.

Supplementary Material 1.

## Data Availability

No datasets were generated or analysed during the current study.
