# Supplementary material for: Morphometric and signal intensity benchmarks of 3D CRANI MR neurography sequence for extraforaminal cranial and occipital nerves visualization: a pilot study
Source: Surg Radiol Anat. 2025 Sep 29;47(1):216. doi: 10.1007/s00276-025-03726-5 (PMC12479606; doi:10.1007/s00276-025-03726-5)
Supplement: Supplementary file 1 — Supplementary Material 1. [file 276_2025_3726_MOESM1_ESM.docx]

**Supplemental figures**

**
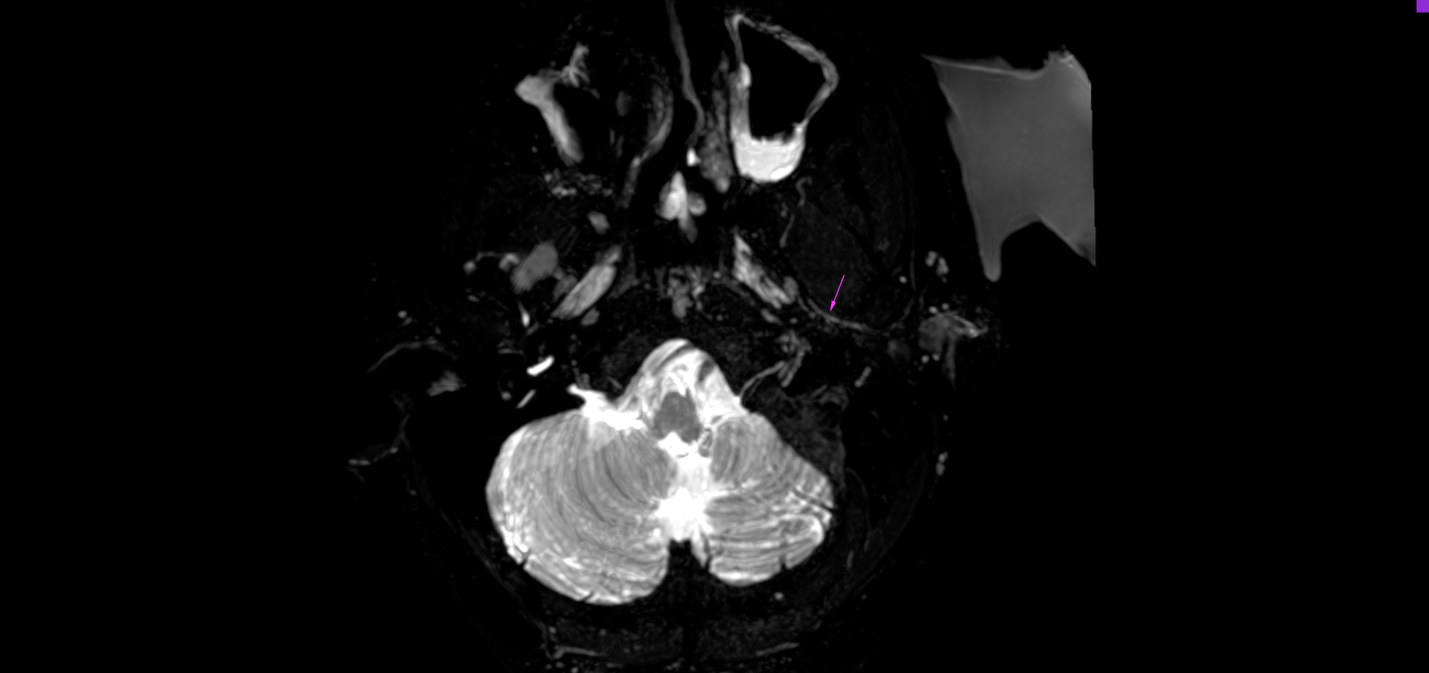
**

**Fig. 1a** Grayscale image of the auriculotemporal nerve (left).

**
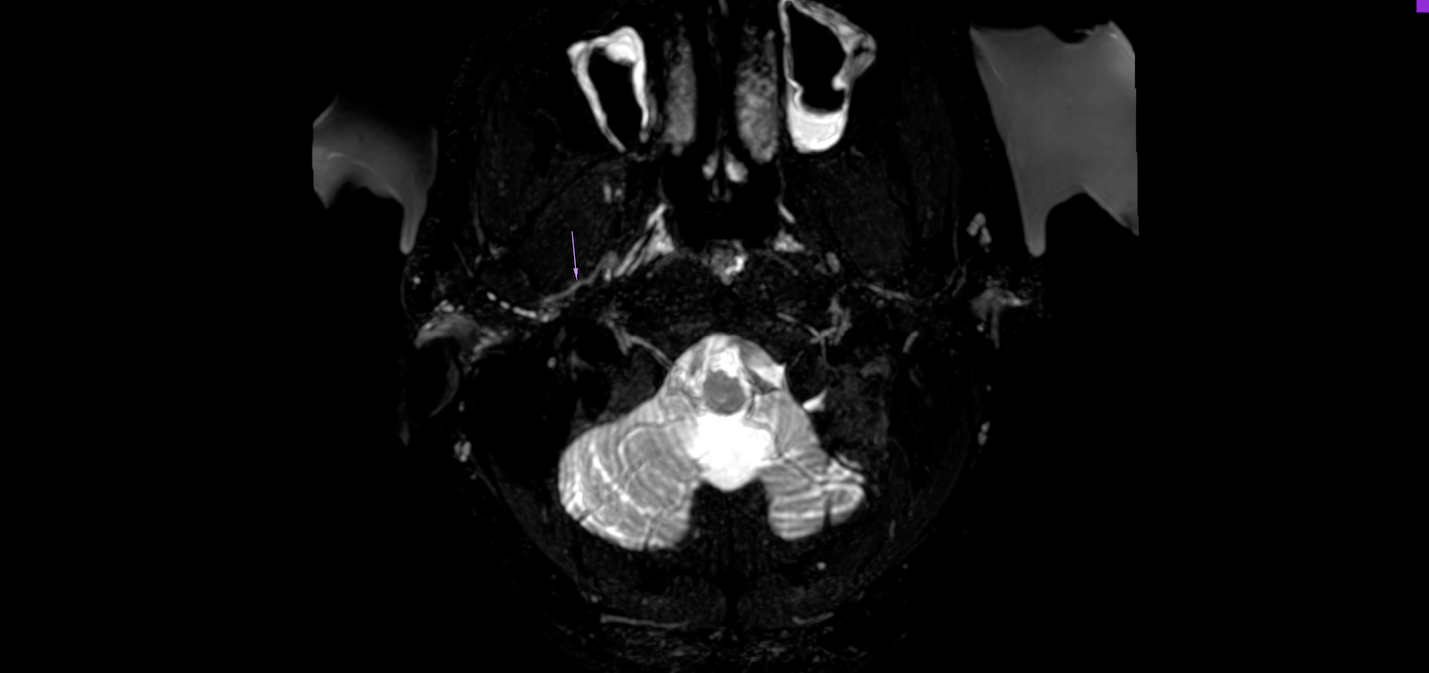
**

**Fig. 1b** Grayscale image of the auriculotemporal nerve (right).

**
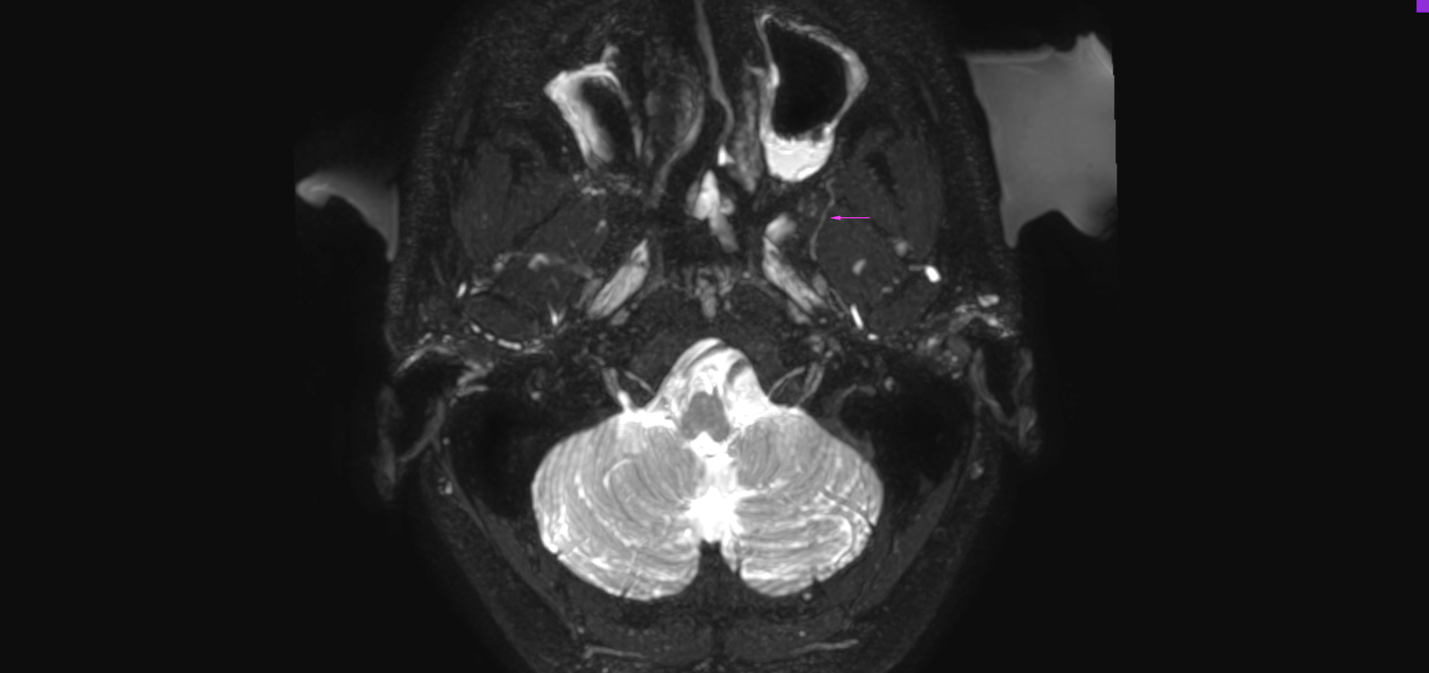
**

**Fig. 2** Grayscale image of the buccal nerve (left).


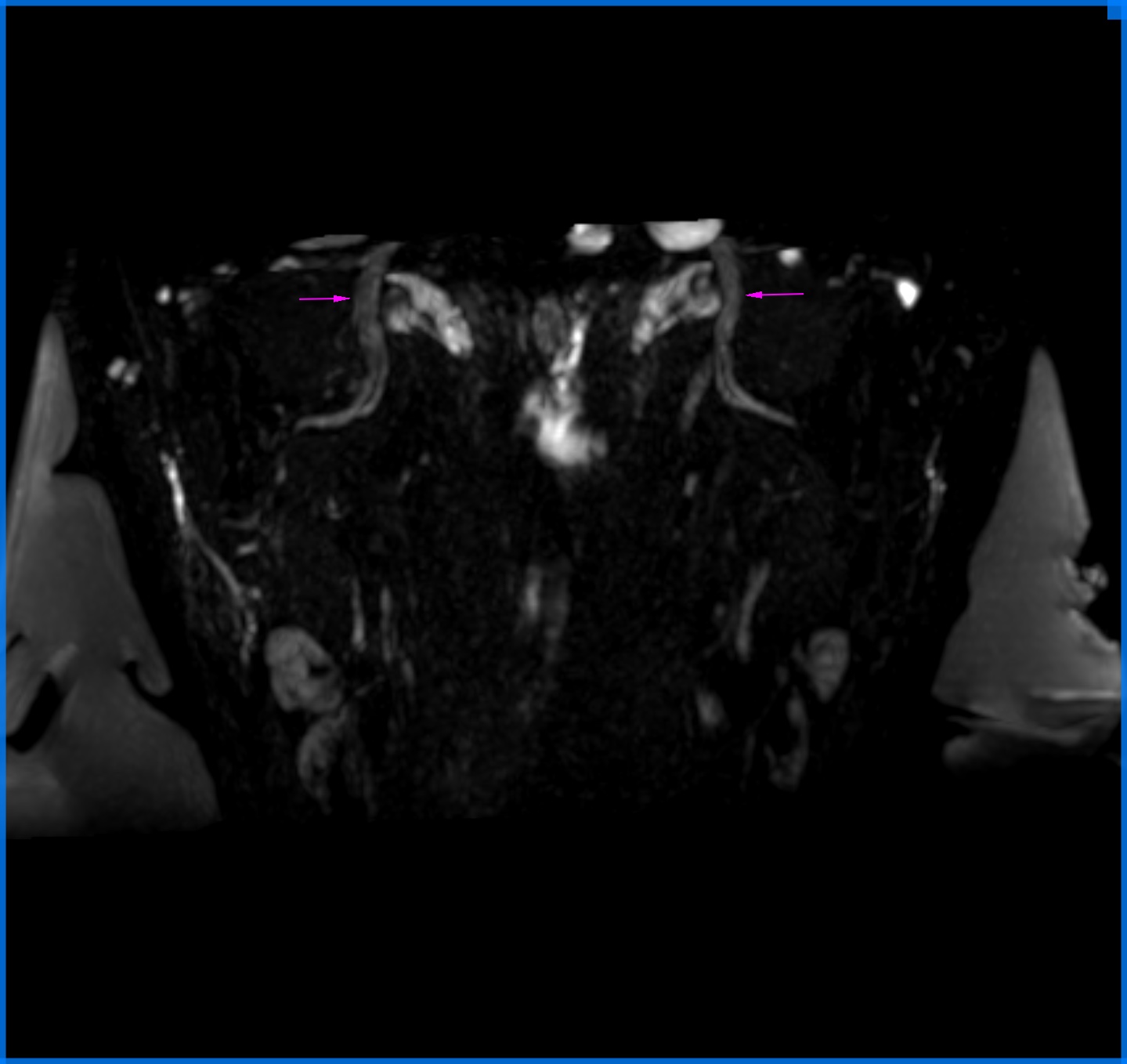


**Fig. 3** Grayscale image of the mandibular nerves


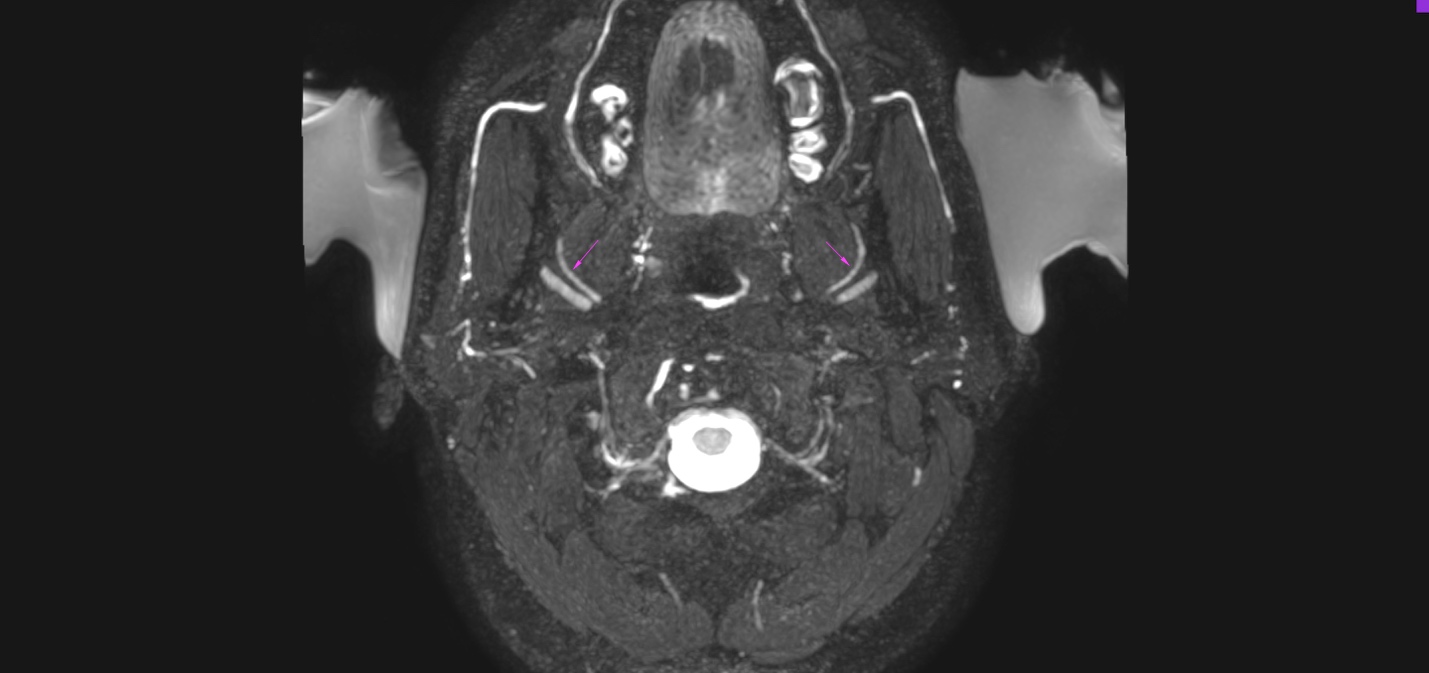


**Fig. 4** Grayscale image of the inguinal nerves.


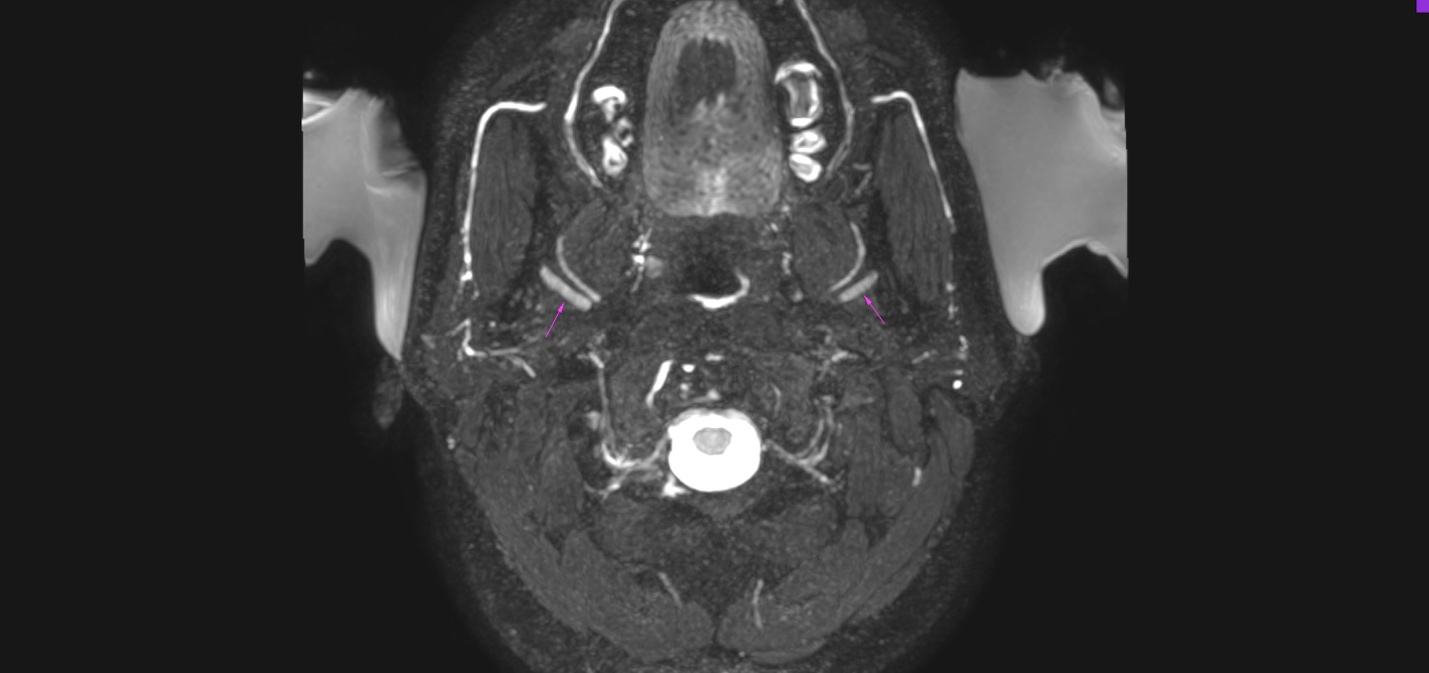


**Fig. 5a** Grayscale image of the proximal parts of the inferior alveolar nerves


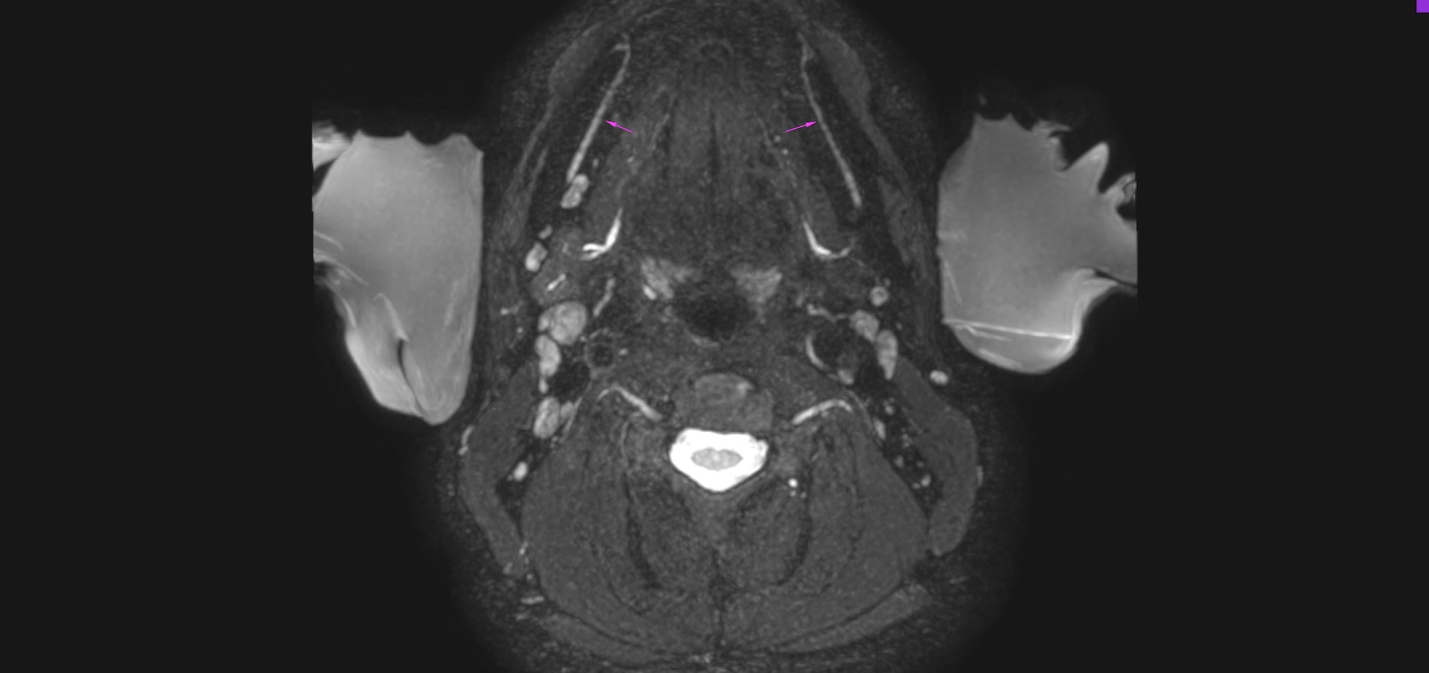


**Fig. 5b** Grayscale image of the distal part of the inferior alveolar nerves


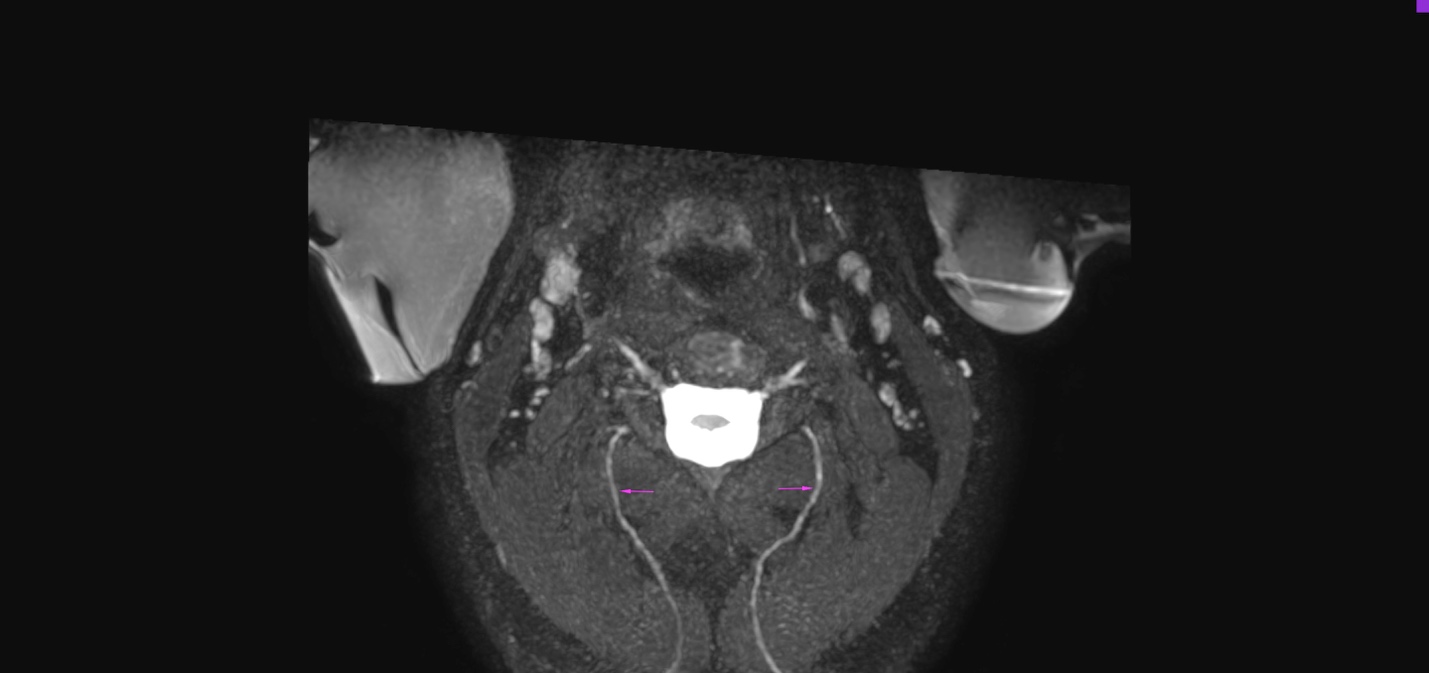


**Fig. 6** Grayscale image of the greater occipital nerves


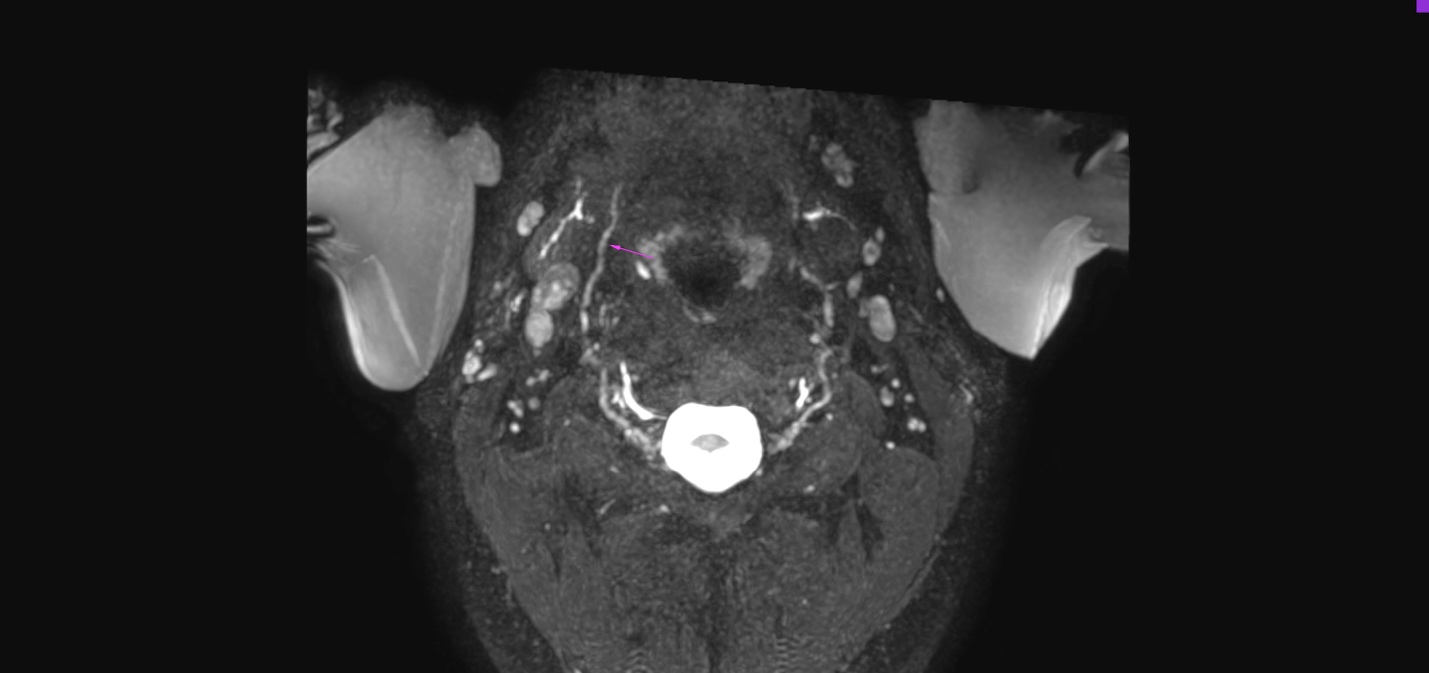


**Fig. 7** Grayscale image of the hypoglossal nerve (right).


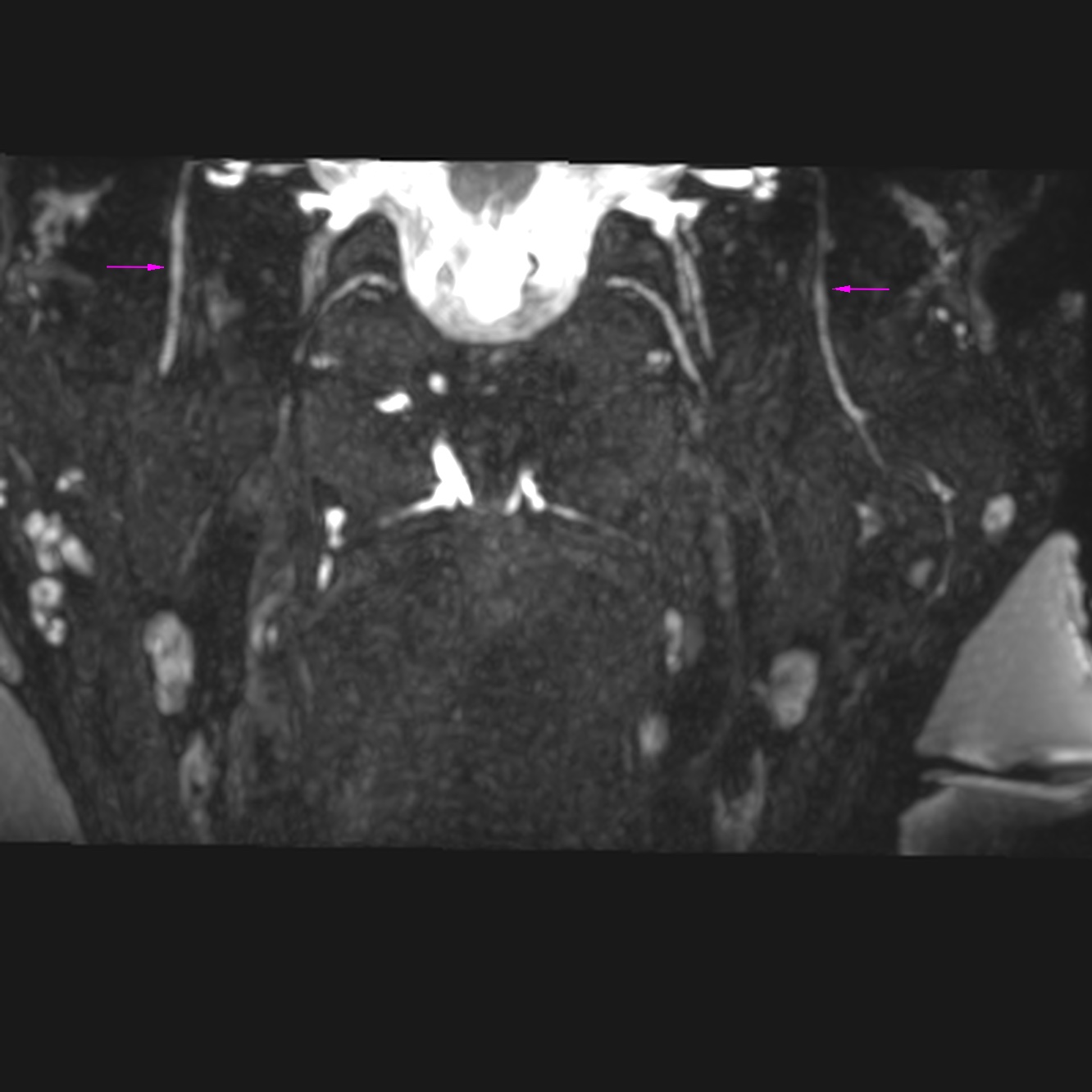


**Fig. 8** Grayscale image of the facial nerves


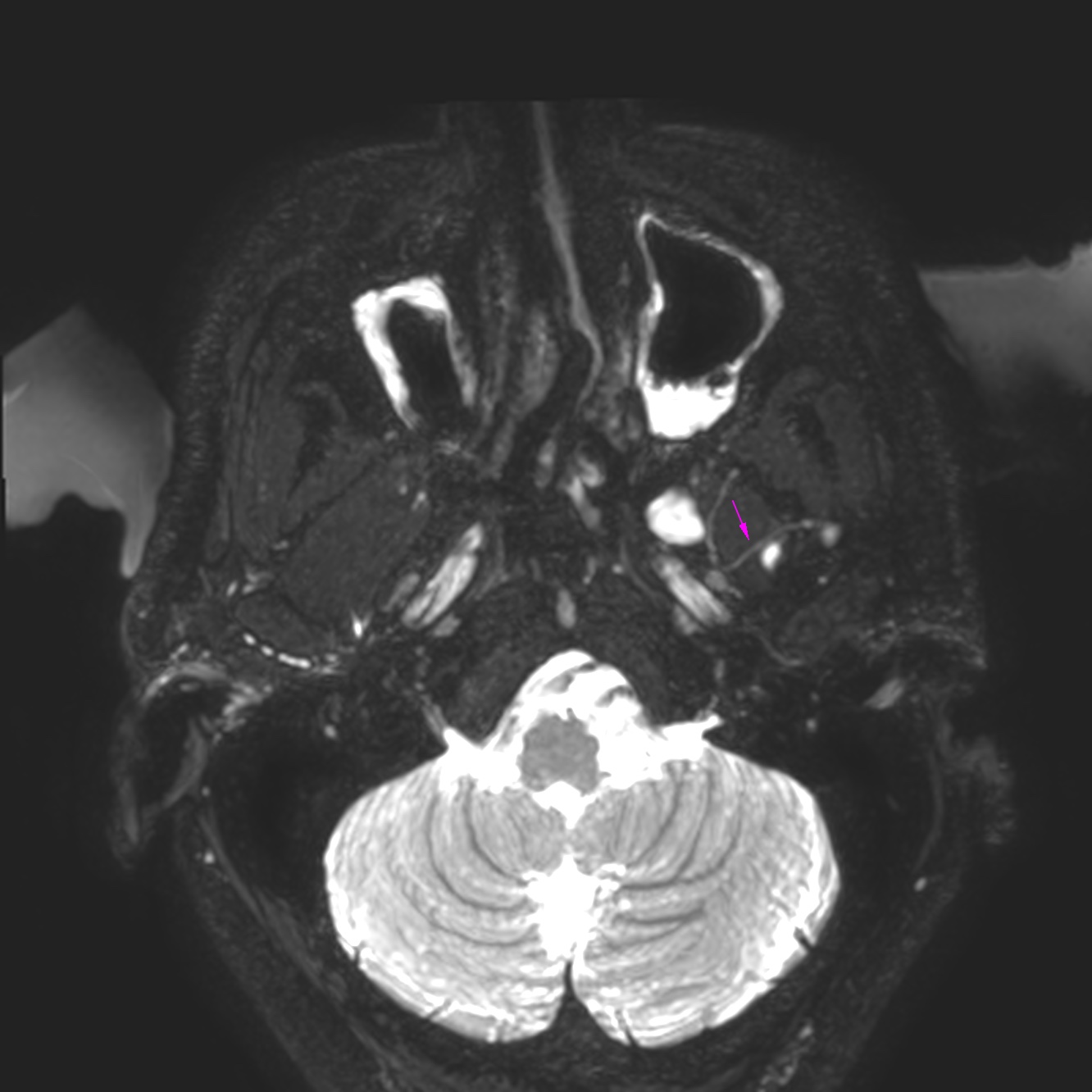


**Fig. 9** Grayscale image of the masseteric nerve (left)


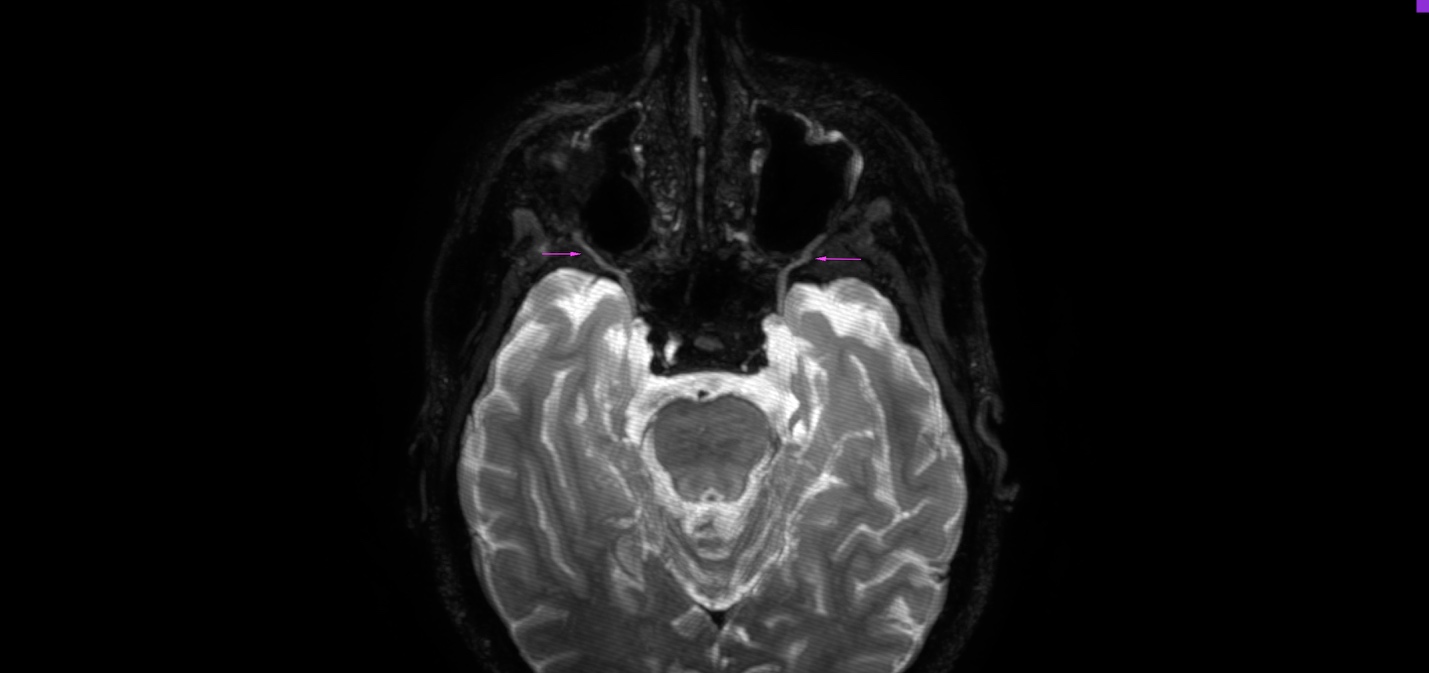


**Fig. 10** Grayscale image of the maxillary nerves
